# Anti-Aging Effect of the Ketone Metabolite β-Hydroxybutyrate in *Drosophila* Intestinal Stem Cells

**DOI:** 10.3390/ijms21103497

**Published:** 2020-05-15

**Authors:** Joung-Sun Park, Yung-Jin Kim

**Affiliations:** 1 Korea Nanobiotechnology Center, Pusan National University, Busan 46241, Korea; 2 Department of Molecular Biology, Pusan National University, Busan 46241, Korea; yjinkim@pusan.ac.kr

**Keywords:** *Drosophila* midgut, β-hydroxybutyrate, intestinal stem cell, centrosome amplification, Niche, DNA damage, heterochromatin stability, aging

## Abstract

Age-related changes in tissue-resident adult stem cells may be closely linked to tissue aging and age-related diseases, such as cancer. β-Hydroxybutyrate is emerging as an important molecule for exhibiting the anti-aging effects of caloric restriction and fasting, which are generally considered to be beneficial for stem cell maintenance and tissue regeneration. The effects of β-hydroxybutyrate on adult stem cells remain largely unknown. Therefore, this study was undertaken to investigate whether β-hydroxybutyrate supplementation exerts beneficial effects on age-related changes in intestinal stem cells that were derived from the *Drosophila* midgut. Our results indicate that β-hydroxybutyrate inhibits age- and oxidative stress-induced changes in midgut intestinal stem cells, including centrosome amplification (a hallmark of cancers), hyperproliferation, and DNA damage accumulation. Additionally, β-hydroxybutyrate inhibits age- and oxidative stress-induced heterochromatin instability in enterocytes, an intestinal stem cells niche cells. Our results suggest that β-hydroxybutyrate exerts both intrinsic as well as extrinsic influence in order to maintain stem cell homeostasis.

## 1. Introduction

Adult stem cells play a key role in tissue homeostasis and regeneration based on their ability to sustain self-renewal and produce differentiated cells [1,2,3,4]. Age-related changes in adult stem cells are closely involved with tissue aging and age-related diseases, including cancer [5,6,7,8,9]. It is well documented that the microenvironmental niche affects age-related changes in adult stem cells, which is a heterogeneous cell population that surrounds the stem cells [9,10]. Hence, studies need to focus on stem cells and their microenvironments in order to elucidate the mechanisms that slow down or recover age-related changes in adult stem cells.

Organismal diet is emerging as an important regulator of adult stem cell function [11]. Caloric restriction and fasting are commonly associated with extended lifespan, delayed onset of age-related diseases, and reduced cancer incidence, and they are generally beneficial for stem cell maintenance and tissue regeneration [12,13]. Recently, the ketone body β-hydroxybutyrate (β-HB) has emerged as an important molecule for imparting the anti-aging effects of caloric restriction and fasting [14].

Over the last decade, ketone bodies (including β-HB) have been studied for their beneficial outcomes in age-related diseases, such as neurodegenerative disorders and cancer [15,16]. Ketone bodies are small molecules that are synthesized in the liver from fats during fasting, prolonged exercise, or under conditions of restricted carbohydrate supply [17,18]. β-HB is first converted to acetyl-CoA, and subsequently to ATP [17,18]. It has recently been reported that β-HB is not only a passive carrier of energy, but it also has a variety of signaling functions that affect the epigenetic state and other activities [18]. The catabolism of β-HB increases the intracellular acetyl-CoA levels that affect mitochondrial and nuclear protein acetylation [17]. The inhibition of histone deacetylase (HDAC) activity is one of the signaling functions of β-HB that regulates longevity and pathways related to diseases of the aging [17]. *Drosophila* species that are heterozygous for a null or hypomorphic Rpd3 (fly homolog of mammalian class I HDACs) allele show a 30–40% increase in their life span [19]. In mammals, β-HB induces the transcription of *FOXO3a* via the inhibition of class I and IIa HDACs [20]. FOXO transcription factors induce the expressions of enzymes that are required for free radical detoxification [20]. Therefore, β-HB utilization affects the mitochondrial redox state, and reduces the production of free radicals [21]. It is also reported that β-HB supplementation extends the longevity in *C. elegans* [22]. From the diversity of age-associated diseases and pathways affected by β-HB signaling, it has been suggested that β-HB derived therapies are promising for broadly enhancing the health span and resilience in humans [23]. The preventive and therapeutic potential of β-HB for age-related diseases (including cancer) might be associated with its action on age-related changes in tissue-resident adult stem cells. However, the effects of β-HB on age-related changes in stem cells remain unexplored.

The *Drosophila* midgut is an excellent model for studying age-related changes of adult stem cells, due to easy genetic manipulation and short lifespan [5,6,7,8,9]. As several studies have revealed the gut-brain axis, research into the intestine is becoming more pronounced [24]. *Drosophila* intestinal stem cells (ISCs) are the only mitotic cells within the adult fly’s midgut [2,3,4], and they are able to generate two types of differentiated cells: absorptive polyploid enterocytes (ECs) and secretory enteroendocrine cells (EEs), via the enteroblasts (EBs) [4]. These cell types can be distinguished by examining the expression levels of cell-specific markers [2,3,4]. ISC proliferation is activated by the intrinsic and extrinsic oxidative stresses that are caused by aging, infection, and high metabolic rate [6,7,8,25,26,27]. In aged and oxidative stressed guts, there is hyperproliferation, DNA damage accumulation, and increased centrosome amplification, which are hallmarks of cancer [7,8,28,29,30,31]. Numerous studies have reported the intrinsic and extrinsic pathways that are associated with age-related changes in ISCs [4,7,32,33,34,35,36,37,38,39,40,41,42,43,44,45,46]. The results of these previous studies strongly suggest that the *Drosophila* midgut is an excellent model system for application in the study of age-related changes in stem cells.

In the present study, we investigated whether β-HB supplementation exerts any beneficial effects on age-related changes of ISCs in the *Drosophila* midgut.

## 2. Results

### 2.1. Inhibitory Effect of β-HB on Age- and Oxidative Stress-Related Centrosome Amplification in Drosophila Midgut ISCs

**Figure 1:**
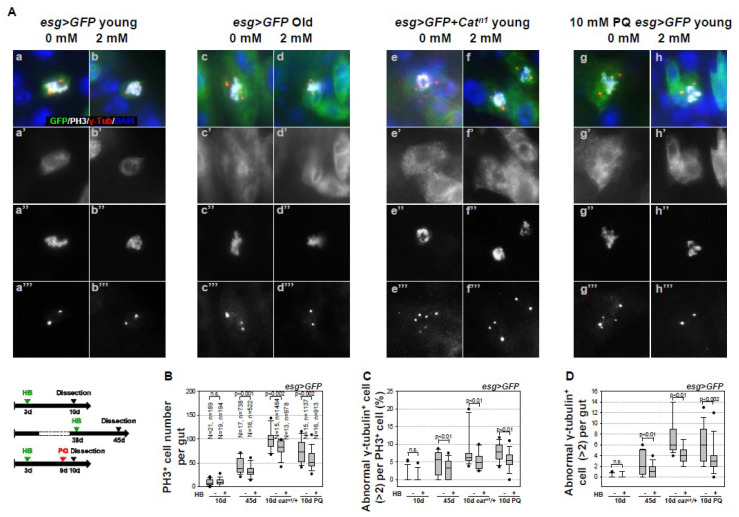
Inhibitory effect of β-HB on age- and oxidative stress-related centrosome amplification in midgut ISCs. (**A**) Guts from 10-day-old *esg>GFP* flies (a–b”’), 45-day-old *esg > GFP* flies (c–d”’), and 10-day-old *esg > GFP* + *Cat^n1^* mutant flies (e–f’”), without (a–a’”, c–c’’’, e–e’’’) or with (b-b’’’, d-d’’’, f-f’’’) 2 mM β-HB feeding for seven days, were stained with anti-γ-tubulin (red), anti-PH3 (white), anti-GFP (green), and DAPI (blue). Ten-day-old *esg > GFP* flies, without (g–g’”) or with (h–h’”) 2 mM β-HB feeding for six days, were treated with 10 mM PQ in standard media for 20 h, after which their guts were stained with anti-γ-tubulin (red), anti-PH3 (white), anti-GFP (green), and DAPI (blue). a’, b’, c’, d’, e’, f’, g’, and h’ indicate enlarged GFP stained images. a”, b”, c”, d”, e”, f”, g”, and h” indicate enlarged PH3 stained images. a”’, b’”, c’”, d’”, e’”, f’”, g’”, and h’” indicate enlarged γ-tubulin stained images. Original magnification is 400×. (**B**) The number of PH3-positive cells was counted in whole guts from 10-day-old *esg > GFP*, 45-day-old *esg > GFP*, 10-day-old *esg > GFP* + *Cat^n1^* mutant, and 10-day-old PQ-treated *esg > GFP* flies, with or without β-HB feeding for seven days. N is the number of observed guts, and n is the number of observed PH3-positive cells. n.s. indicates not significant (p>0.05). (**C**) The frequency of supernumerary centrosomes (>2) per mitotic ISC in 10-day-old *esg > GFP*, 45-day-old *esg > GFP*, 10-day-old *esg > GFP* + *Cat^n1^* mutant, and 10-day-old PQ-treated *esg > GFP* flies, with or without β-HB feeding for seven days. The centrosome numbers were counted in mitotic ISCs (PH3-and GFP-positive cells) in the midgut. n.s. indicates not significant (*p* > 0.05). (**D**) The frequency of mitotic ISCs with supernumerary centrosomes per gut in 10-day-old *esg > GFP*, 45-day-old *esg > GFP*, 10-day-old *esg > GFP* + *Cat^n1^* mutant, and 10-day-old PQ-treated *esg > GFP* flies, with or without β-HB feeding for seven days. The error bar represents standard error. *p*-values were calculated using Student’s *t*-test. n.s. indicates not significant (*p* > 0.05).

Age- and oxidative stress-related centrosome amplification with supernumerary centrosomes has been reported in ISCs. [30,47]. In this study, we investigated the effect of β-HB on age-related centrosome amplification in ISCs of the gut from *esg* > *green fluorescent protein (GFP)* flies, which marks only ISC/EB [2]. ISCs were stained with anti-γ-tubulin (a centrosome marker), anti-PH3 (a marker of mitotic ISCs), and anti-GFP (a marker of ISCs/EBs), and the number of cells displaying centrosome amplification was assessed. Supernumerary centrosomes were observed in 5.69% of mitotic ISCs in 45-day-old *esg* > *GFP* flies (Figure 1A(c–c’’’),C) and in 7.41% 10-day-old *esg* > *GFP* + *Cat^n1^* flies (Figure 1A(e–e’’’),C), a model of intrinsic oxidative stress (Choi et al., 2008) [7], as compared to 1.05% in 10-day-old *esg* > *GFP* flies (Figure 1A(a–a’’’),C). The number of mitotic ISCs with supernumerary centrosomes per gut was 2.47 in 45-day-old *esg* > *GFP* flies and 7.33 in 10-day-old *esg* > *GFP* + *Cat^n1^* flies, as compared to 0.09 in 10-day-old *esg* > *GFP* flies (Figure 1D). These results indicate that, with aging and subsequent to oxidative stress exposure, there is an increase in centrosome amplification in midgut ISCs. Interestingly, β-HB treatment reduces the age- and oxidative stress-related increase in the number of PH3-positive cells (Figure 1B). β-HB treatment reduces age- and oxidative stress-related increase of supernumerary centrosomes in 3.64% of mitotic ISCs in 45-day-old *esg* > *GFP* flies (Figure 1A(d–d’’’),C) and in 5.01% of mitotic ISCs in 10-day-old *esg* > *GFP* + *Cat^n1^* flies (Figure 1A(f–f’’’),C). The number of mitotic ISCs with supernumerary centrosomes per gut was reduced by 1.19 in 45-day-old *esg* > *GFP* flies, and by 4.08 in 10-day-old *esg* > *GFP* + *Cat^n1^* flies, whereas no change was observed in 10-day-old *esg* > *GFP* flies (Figure 1D). We applied PQ treatment as extrinsic oxidative stress to confirm whether β-HB inhibits oxidative stress-induced centrosome amplification in the midgut. Briefly, three-day-old *esg* > *GFP* flies with or without 2 mM β-HB treatment for six days were treated with 10 mM paraquat (PQ) for 18 h. Mitotic ISCs (9 to 75.8) and mitotic ISCs with supernumerary centrosomes (1.06% to 8.18%) were both observed to increase in the PQ-treated *esg* > *GFP* flies (Figure 1A(g–g’’’),B,C), as compared to control flies (Figure 1A(a–a’’’),B,C). Furthermore, there was an increase in the number of mitotic ISCs with supernumerary centrosomes per after PQ treatment (0.09 to 6.2; Figure 1D). However, in β-HB pre-treated *esg* > *GFP* flies, the number of PH3-positive cell (75.8 to 57.06), mitotic ISCs with supernumerary centrosomes (8.18% to 6.13%), and the number of mitotic ISCs with supernumerary centrosomes per gut (6.2 to 3.5) were decreased after PQ treatment (Figure 1A(h–h’’’),B,C). The result from *esg* > *GFP* flies indicate that β-HB can reduce age- and oxidative stress-induced centrosome amplification in adult intestinal stem cells in vivo.

In addition, we confirmed the inhibitory effect of β-HB on age-related centrosome amplification in wild type fly, as shown in Figure S1 and the Legend in Supplementary Materials (Figure S1). These results indicate that β-HB strongly suppresses age- and oxidative stress-induced centrosome amplification in *Drosophila* midgut ISCs.

### 2.2. Inhibitory Effect of β-HB on Age-Related Increases in DNA Damage Accumulation in Midgut ISCs

**Figure 2:**
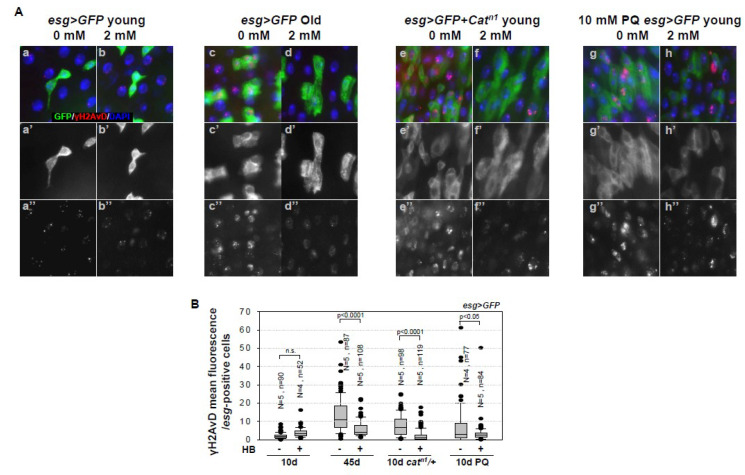
β-HB inhibits age- and oxidative stress-induced DNA damage accumulation in midgut intestinal stem cells (ISCs) and progenitors. (**A**) Guts from 10-day-old *esg > GFP* flies (a–b”), 45-day-old *esg>GFP* flies (c–d”), and 10-day-old *esg > GFP* + *Cat^n1^* mutant flies (e–f”), without (a–a”, c–c’’, e–e’’) or with (b–b’’, d–d’’, f–f’’) 2 mM β-HB feeding for seven days, were stained with anti-γH2AvD (red), anti-GFP (green), and DAPI (blue). Ten-day-old *esg>GFP* flies, without (g–g”) or with (h–h”) 2 mM β-HB feeding for six days, were treated with 10 mM PQ in standard media for 20 h, after which their guts were stained with anti-γH2AvD (red), anti-GFP (green), and DAPI (blue). a’, b’, c’, d’, e’, f’, g’, and h’ indicate enlarged GFP stained images. a”, b”, c”, d”, e”, f”, g”, and h” indicate enlarged γH2AvD stained images. Original magnification is 400×. (**B**) Graph showing the average fluorescence intensity of γH2AvD in GFP-positive cells in 10-day-old *esg > GFP*, 45-day-old *esg > GFP*, 10-day-old *esg > GFP* + *Cat^n1^* mutant, and 10-day-old PQ-treated *esg > GFP* flies, with or without β-HB feeding for seven days. N is the number of observed guts and n is the number of observed cells. The error bar represents standard error. *p*-values were calculated using Student’s *t*-test. n.s. indicates not significant (*p* > 0.05).

Centrosome amplification is associated with cell cycle arrest due to DNA damage, particularly during the G2-M phase [47,48]. In this study, we assessed the correlation between DNA damage and centrosome amplification, while considering the inhibitory effects of β-HB. Previous studies have reported the age- and oxidative stress-induced increases of DNA damage accumulation in *Drosophila* midgut ISCs [28,29]. Based on these studies, we undertook examining whether β-HB treatment reduces the age- and oxidative stress-induced DNA damage accumulation. This study confirmed the presence of age- and oxidative stress-induced increases of DNA damage accumulation in midgut ISCs after aging and oxidative stress exposure. Briefly, the cells were stained with anti-γ-H2AvD (a DNA damage marker) and anti-GFP (diploid cells marker, for both ISCs and EBs). The level of γH2AvD fluorescence in *esg*-positive cells increased in 45-day-old *esg* > *GFP* flies (13.31, Figure 2A(c–c’’),B),in 10-day-old *esg > GFP* + *Cat^n1^* flies (7.68, Figure 2A(e–e’’),B), and in PQ-treated *esg > GFP* flies (4.03, Figure 2A(g–g’’),B), as compared to 10-day-old *esg > GFP* flies (2.12, Figure 2A(a–a’’),B). As expected, β-HB treatment resulted in reduced DNA damage accumulation in *esg*-positive cells of 45-day-old *esg > GFP* flies (13.31 to 5.97, Figure 2A(d–d’’),B), 10-day-old *esg > GFP* + *Cat^n1^* flies (7.68 to 2.15, Figure 2A(f–f’’),B), and PQ-treated *esg > GFP* flies (7.29 to 3.28, Figure 2A(h–h’’),B), as compared to the non-treated flies (Figure 2A(c–c’’, e–e’’, g–g’’),B). These results indicate that β-HB treatment decreased age- and oxidative stress-related increases of DNA damage accumulation in ISCs.

### 2.3. Inhibitory Effect of β-HB on Age-Related Loss of Heterochromatin Stability in Midgut ECs, ISC Niche Cells

**Figure 3:**
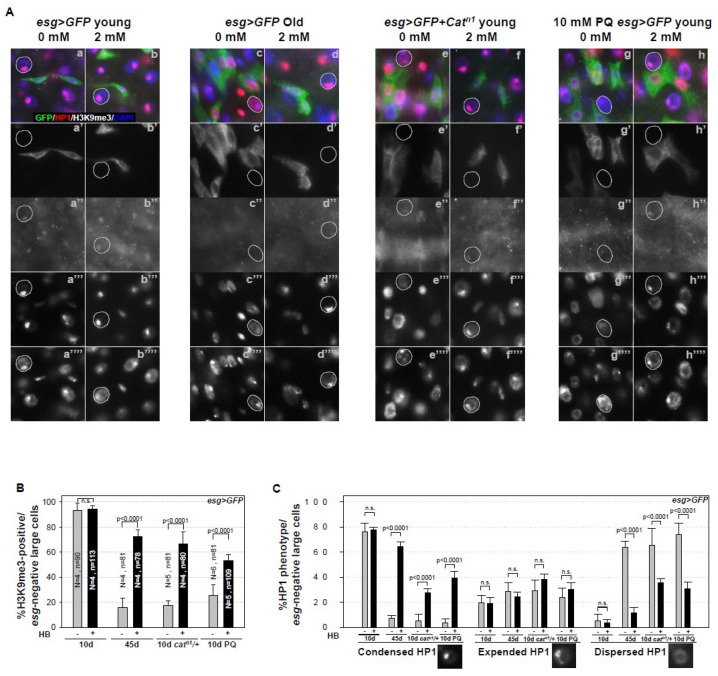
β-HB inhibits age- and oxidative stress-induced heterochromatin instability in midgut ECs. (**A**) Guts from 10-day-old *esg > GFP* flies (a–b”’’), 45-day-old *esg > GFP* flies (c–d”’’), and 10-day-old *esg > GFP* + *Cat^n1^* mutant flies (e–f’’”), without (a–a’’”, c–c’’’’, e–e’’’’) or with (b–b’’’’, d–d’’’’, f–f’’’’) 2 mM β-HB feeding for seven days, were stained with anti-HP1 (red), anti-H3K9me3 (white), anti-GFP (green), and DAPI (blue). Ten-day-old *esg > GFP* flies, without (g–g’’”) or with (h–h’’”) 2 mM β-HB feeding for six days, were treated with 10 mM PQ in standard media for 20 h, after which their guts were stained with anti-HP1 (red), anti-H3K9me3 (white), anti-GFP (green), and DAPI (blue). a’, b’, c’, d’, e’, f’, g’, and h’ indicate enlarged GFP stained images. a”, b”, c”, d”, e”, f”, g”, and h” indicate enlarged H3K9me3 stained images. a”’, b’”, c’”, d’”, e’”, f’”, g’”, and h’” indicate enlarged HP1 stained images. a”’’, b’’’’, c’’”, d’’”, e’’”, f’’”, g’’”, and h’’” indicate enlarged DAPI stained images. White dotted circles indicate the nuclei of ECs (*esg*-negative cell). Original magnification is 400×. (**B**) Graph showing the proportion of H3K9me3-positive cells in GFP-negative large cells (ECs) in 10-day-old *esg* > *GFP*, 45-day-old *esg* > *GFP*, 10-day-old *esg* > *GFP* + *Cat^n1^* mutant, and 10-day-old PQ-treated *esg* > *GFP* flies, with (black bar) or without (gray bar) β-HB feeding for seven days. (**C**) Graph showing the proportion of condensed, expanded, and dispersed HP1 phenotype in GFP-negative large cells in 10-day-old *esg* > *GFP*, 45-day-old *esg* > *GFP*, 10-day-old *esg* > *GFP* + *Cat^n1^* mutant, and 10-day-old PQ-treated *esg* > *GFP* flies, with (black bar) or without (gray bar) β-HB feeding for seven days. N is the number of observed guts, and n is the number of observed cells. The error bar represents standard error. *p*-values were calculated using Student’s *t*-test. n.s. indicates not significant (*p* > 0.05).

A previous study reported the age- and oxidative stress-related loss of heterochromatin stability due to the loss and dispersion of tri-methylated histone H3 Lys 9 (H3K9me3) and heterochromatin protein1 (HP1) in midgut ECs, as niche aging [49]. HP1 is a crucial heterochromatin component that binds H3K9me3 [50]. We examined the age- and oxidative stress-induced increases of heterochromatin instability in ECs after aging and oxidative stress exposure while using anti-H3K9me3 and anti-HP1 antibodies. Single-spotted H3K9me3 foci in the nuclei of ECs (*esg*-negative large cells) were clearly detected in 10-day-old *esg* > *GFP* flies (Figure 3A(a–a’’)). In 45-day-old *esg* > *GFP* (93.05% to 15.76%, Figure 3A(c–c’’),B), 10-day-old *esg* > *GFP* + *Cat^n1^* (93.05% to 17.46%, Figure 3A(e–e’’),B), and PQ-treated *esg* > *GFP* (93.05% to 25.18%, Figure 3A(g–g’’),B) flies, single-spotted H3K9me3 foci were significantly deceased in the EC nuclei. Following β-HB treatment, age- and oxidative stress-related loss of H3K9me3 foci in the nuclei of ECs was recovered in 45-day-old *esg* > *GFP* flies (15.76% to 72.2%, Figure 3A(d–d’’),B), 10-day-old *esg* > *GFP* + *Cat^n1^* flies (17.46% to 66.37%, Figure 3A(f–f’’),B), and PQ-treated *esg* > *GFP* flies (25.18% to 53.16%, Figure 3A(h–h’’),B), as compared to non-treated flies (Figure 3A(c–c’’, e–e’’, g–g’’)). These data indicate that the age-dependent diminishment of H3K9me3 can be rescued by β-HB treatment. Simultaneously, we investigated the effect of β-HB on age-related changes of HP1 in midgut ECs. We categorized HP1 phenotypes into three types, designated as condensed (single-spotted and strong signal), expanded (single-spotted but branched or ring-shaped, and weak signal), and dispersed (dispersed throughout the nucleus, or almost no signal), to quantify HP1 signals in ECs. Single- and condensed-spotted HP1 in the nucleus of ECs (*esg*-negative large cells) was clearly detected in 10-day-old *esg* > *GFP* flies (Figure 3A(a–a’’’)). This is consistent with age-related loss, expansion, or dispersion of HP1 detected in ECs (*esg*-negative large cells), when compared to control flies (Figure 3A(c–c’’’, e–e’’’, g–g’’’)). Age- and oxidative stress-related losses of HP1 in the ECs nuclei were recovered by β-HB treatment in 45-day-old *esg* > *GFP* (Figure 3A(d–d’’’)), 10-day-old *esg* > *GFP* + *Cat^n1^* flies (Figure 3A(f–f’’’)), and PQ-treated esg > *GFP* flies (Figure 3A(h–h’’’)), as compared to the non-treated flies (Figure 3A(c–c’’’, e–e’’, g–g’’’)). Following β-HB treatment, age- and oxidative stress-related decrease of the proportion of condensed HP1 type in ECs was significantly recovered in 45-day-old *esg* > *GFP* flies, 10-day-old *esg* > *GFP* + *Cat^n1^* flies, and PQ-treated *esg* > *GFP* flies, as compared to the non-treated flies (Figure 3C). These results indicate that β-HB treatment markedly rescues age- and oxidative stress-induced loss or dispersion of HP1 in ECs. These results suggest that age- and oxidative stress-related increases of heterochromatin instability in ECs, a niche aging, can be decreased by β-HB treatment.

## 3. Discussion

In the present study, we report that β-HB, a key molecule that is involved in ketone body signaling, has an inhibitory effect on adult stem cell aging in the *Drosophila* midgut. The key results of this study are: (1) β-HB inhibits age- and oxidative stress-induced increases of centrosome amplification in midgut ISCs (centrosomes amplification are reported to be an early event in tumorigenesis and senescence), (2) β-HB inhibits age- and oxidative stress-induced DNA damage accumulation in midgut ISCs and progenitors, and (3) β-HB inhibits age- and oxidative stress-induced increases of heterochromatin instability in midgut ECs, ISC niche cells.

We showed that β-HB reduces excessive centrosome amplification in *Drosophila* midgut ISCs undergoing aging and intrinsic (*Cat^n1^* mutant) and extrinsic (PQ) oxidative stresses (Figure 1, Figure S1). Studies have correlated centrosome abnormalities with the presence of tumorigenesis and tumor progression [47], which are reported to interfere with asymmetric stem cell division, which leads to increased stem cell population hyperplasia [47]. In addition, we determined that β-HB decreases not only the centrosome amplification, but also age- and oxidative stress-induced increases of ISC proliferation. It has been reported that age- or oxidative stress-related centrosome amplification are associated with PVR, EGFR, and AKT/TOR signaling [30,39]. Several signaling pathways, including Wnt [43,51,52], JAK/STAT [53], EGFR [33,43,54,55], Hippo [35,36], PVR [7,8,34], and insulin receptor [38,45] pathways, regulate ISC proliferation. Furthermore, some studies have shown that age- and oxidative stress-related increases in ISC proliferation are regulated by the PVR-p38 [7,8], JNK-EGFR-FOS [26,37,49], Wnt-Myc pathways [51], and insulin/IGF1 [56] and AKT/TOR signaling [30,39]. When considering these results, future research examining the relationship between β-HB and these signaling pathways, especially AKT/TOR, in ISC proliferation and centrosome amplification is required.

Our results further indicate that β-HB treatment reduces the age- and oxidative stress-induced DNA damage accumulation in midgut ISCs and progenitors, when considering the changes that were obtained in the fluorescence intensity of γH2AvD (the phosphorylated *Drosophila* histone variant of H2A on Ser137, homologous to mammalian γH2AX) (Figure 2). GammaH2AX is a well-established marker of DNA damage [57], and an irradiation-induced γH2AvD increase in ISCs indicates that the γH2AvD signal in ISCs does indicate the presence of DNA damage in *Drosophila* [58]. We have previously reported that an increase in γH2AvD is a key feature of *Drosophila* ISC aging and the changes in γH2AvD is closely associated with the age-related accumulation of 8-oxo-2′-deoxyguanosine (a well-known product of ROS-induced DNA damage) [28]. β-HB induces an increase in the levels of the ROS scavengers, viz., catalase, and SOD [59]. More recently, β-HB has been shown to prevent cell senescence and exert anti-aging effects through the upregulation of Oct4 and Lamin B1, which are key factors acting against DNA damage [14]. In the current study, we show an additional benefit of β-HB, in its ability to protect against DNA damage accumulation in stem cells.

Our results further indicate that β-HB treatment recovers the age- and oxidative stress-induced loss of heterochromatin stability consequent to the loss and dispersion of H3K9me3 and HP1 in midgut ECs (Figure 3). It is well known that ECs surround ISCs [2,3] and they are a major source of niche signal on ISC self-renewal [60]. ECs are constantly exposed to external stresses, such as injury, infection, or damaged conditions, resulting in ECs death, which is a major cause of accelerated ISC proliferation [25,31,61,62]. A previous study reported that age-related loss of heterochromatin stability in differentiated ECs is associated with an increase of age-related phenotypes of ISC (hyperproliferation and DNA damage accumulation) through apoptotic death, as a niche aging [49]. These observations indicate that β-HB acts as an anti-aging agent in the adult fly midgut ISCs directly via the reduction of centrosome amplification and DNA damage accumulation, and it also affects niche survival via increased heterochromatin stability. When considering the previous study results, it is likely that β-HB is intrinsically as well as extrinsically involved in the maintenance of adult ISCs. In a previous study, we also showed that AKT/TOR signaling induces DNA damage accumulation in *Drosophila* ISCs/EBs [29]. Another study reported that TOR activity in ECs increases with age, and the increased AKT/TOR signaling in ECs is related to the loss of heterochromatin stability in ECs [49]. It is well documented that the inhibition of mTOR is directly implicated in lifespan extension [9,10]. β-HB is known to inhibit mTOR signaling in intestinal cells [63]. Further studies are required to explore whether the inhibitory effects of β-HB on age-related change of ISCs are associated with AKT/TOR signaling. The data of life span in *Drosophila* are also required to know whether β-HB is really effective on aging in both physiological and pathological conditions.

In summary, the data that were obtained in the current study demonstrate that β-HB reduce age- and oxidative stress-induced centrosome amplification, hyperproliferation, and DNA damage accumulation of ISCs, and the loss of heterochromatin stability in ECs, also regarded as the ISC niche cells in the *Drosophila* midgut. Our data suggest that β-HB supplementation has the potential to maintain stem cell homeostasis, both intrinsically and extrinsically.

## 4. Material and Methods

### 4.1. Fly Stock

Fly stocks were maintained at 25 °C on standard food under an approximate 12 h/12 h light/dark cycle. Food consisted of 79.2% water, 1% agar, 7% cornmeal, 2% yeast, 10% sucrose, 0.3% bokinin, and 0.5% propionic acid. In order to avoid larval overpopulation in all vials, 50–60 adult flies per vial were transferred to new food vials every 2–3 days for a period of 50–60 days or longer. The *esg-Gal4,UAS-GFP/CyO* strain was provided by the Drosophila Genetic Resource Center (DGRC, Kyoto, Japan). The *esg > GFP* (*esg-al4,UAS-GFP/+; +/+*) flies were obtained from a cross of Oregon-R males and *esg-Gal4,UAS-GFP/CyO* females. *Oregon-R* flies were used as the wild type. The *Catalase* heterozygous mutant flies (*Cat^n1^* mutant, Bloomington Drosophila Stock Center), a model of intrinsic oxidative stress, is based on previous reports showing a gene dosage-dependent effect on catalase activity [64]. The results described in this study were obtained using female flies.

### 4.2. Immunochemistry

Intact adult guts were dissected and fixed at room temperature. For anti-green fluorescent protein (GFP) antibody staining, the guts were fixed for 1 h in 4% formaldehyde (Sigma–Aldrich, St. Louis, MO, USA). For anti-γH2AvD and Dl antibody staining, the guts were fixed for 30 min in 4% paraformaldehyde (Electron Microscopy Science, USA), dehydrated for 5 min. in 50, 75, 87.5, and 100% methanol, and rehydrated for 5 min. in 50, 25, and 12.5% methanol in PBST (0.1% Triton X-100 in phosphate-buffered saline) for postfixing. After washing with PBST, the samples were incubated for 1 h with secondary antibodies at 25 °C, washed again in PBST, mounted with Vectashield (Vector Laboratories, Burlingame, CA, USA), and then analyzed using a Zeiss Axioskop 2Plus microscope (Carl Zeiss Inc., Göttingen, Germany). The PH3^+^ cells were counted in the entire midgut.

### 4.3. Antisera

The following primary antibodies diluted in PBST were used in these experiments: mouse anti-Dl, mouse anti-Arm (Developmental Studies Hybridoma Bank, Iowa City, IA, USA), 1:200; mouse anti-GFP and rabbit anti-GFP (Molecular Probes, Eugene, OR, USA), 1:1000; rat anti-GFP (Nacalai Tesque Inc., Kyoto. Japan), 1:1000; rabbit anti-γH2AvD (Rockland, Gilbertsville, PA, USA) 1:2000; rabbit anti-phospho-histone H3 (PH3, Millipore, Billerica, MA, USA), 1:1000; mouse anti-γ-tubulin (Sigma–Aldrich), 1:1000; rabbit anti-H3K9me3 (Millipore, Billerica, MA, USA), 1:200; and, mouse anti-HP1 (DSHB, Iowa City, IA, USA), 1:200. The following secondary antibodies diluted in PBST were used: goat anti-rabbit FITC (Jackson ImmunoResearch, West Grove, PA, USA), 1:400; goat anti-rabbit Cy3 (Jackson ImmunoResearch), 1:400; goat anti-mouse FITC (Jackson ImmunoResearch), 1:400; goat anti-mouse Cy3 (Jackson ImmunoResearch), 1:400; goat anti-rat FITC (Jackson ImmunoResearch), 1:400, goat anti-rabbit Alexa Fluor^®^ 647 (Jackson ImmunoResearch), 4′,6-diamidino-2-phenylindole (DAPI, Molecular Probes), 1:1000.

### 4.4. β-Hydroxybutyrate Feeding Assay

β-Hydroxybutyrate (Sigma–Aldrich, St. Louis, MO, USA, working concentration, 2 mM) was added to the standard food media. Three-day-old *esg > GFP*, wild type flies, 38-day-old *esg > GFP*, wild type flies, and 3-day-old *esg > GFP* + *Cat^n1^*, *Cat^n1^*/+ flies were fed 2 mM β-HB in standard media for seven days at 25 °C [18,22]. Every two days, flies were transferred to fresh food vials containing β-HB.

### 4.5. β-Hydroxybutyrate Pre-Feeding Assay

Three-day-old *esg > GFP* or wild type flies treated with 2 mM β-HB for six days [18,22]. After treatment with 10 mM paraquat (PQ, methyl viologen, Sigma–Aldrich) in standard food media for 18–20 h at 25 °C and then guts were analyzed by immunostaining.

### 4.6. Paraquat Feeding Assay

Flies were fed 10 mM PQ in standard media for in standard food media for 18–20 h at 25 °C and then guts were analyzed by immunostaining.

### 4.7. Quantitative Analysis

The number of PH3-positive cells in the whole gut was counted to quantitatively analyze PH3-positive cells. For quantitative analysis of centrosome amplification, we determined the number of γ-tubulin stained spots per PH3-positive cell in the whole midguts. To quantitatively analyze the percentage of H3K9me3 and HP1, individual enterocytes in the posterior midgut from obtained images were cropped to individual image files, and serially analyzed. Each value was obtained as the percentage in a single midgut, and the results are given as the mean of each percentage of midguts. The quantified data are expressed as the mean ± SE. Significant differences were identified while using Student’s *t*-test. Sigma Plot 10.0 (Systat Software Inc., San Jose, CA, USA) was used for analysis of standard error.

### 4.8. Quantification of γH2AvD Fluorescence Levels (Means)

Adobe Photoshop CS6 extended software (Abode System Inc., San Jose, CA, USA) was applied to quantify integrated fluorescence intensities from independent samples for each condition and genotype analyzed. The fluorescence level of foci was measured within the nucleus, whose boundary was defined from the DAPI image. The image fluorescence levels were quantified in view area of the posterior midgut [29].

## Supplementary Materials

Supplementary materials can be found at https://www.mdpi.com/1422-0067/21/10/3497/s1.

## Abbreviations

β-HB: β-Hydroxybutyrate; HDAC, Histone deacetylase; Foxo, Forkhead box; ISC, Intestinal stem cell; EC, Enterocyte; EB, Enteroblast; EE, Enteroendocrine cell; PH3, phosphorylated Histone H3; PQ, Paraquat; GFP, Green fluorescent protein; γH2AvD, phosphorylated *Drosophila* histone variant of H2A (Ser137); HP1, Heterochromatin protein 1; H3K9me3, tri-methyl histone H3 lysine9; AKT, Protein kinase B; TOR, Target of rapamycin; EGFR, Epidermal growth factor receptor; PVR, PDGF- and VEGF-receptor related; SOD, Superoxide dismutase.
