# Supplementary material for: Anti-Aging Effect of the Ketone Metabolite β-Hydroxybutyrate in Drosophila Intestinal Stem Cells
Source: Int J Mol Sci. 2020 May 15;21(10):3497. doi: 10.3390/ijms21103497 (PMC7278929; doi:10.3390/ijms21103497)
Supplement: Supplementary file 1 [file ijms-21-03497-s001.pdf]

Supplementary materials

Inhibitory effect of β-HB on age- and oxidative stress-related centrosome amplification in *Drosophila* midgut ISCs.

We confirmed the inhibitory effect of β-HB on age-related centrosome amplification in wild-type fly. Supernumerary centrosomes were observed in 2.94% of mitotic ISCs in 45-day-old wild-type flies (Figure S1A c-c′′, Figure S1C) and in 9.47% 10-day-old *Cat*^{n1} mutant flies (Fig.S1A e-e′′, Figure S1C), a model of intrinsic oxidative stress (Choi et al., 2008), as compared to 1.16% in 10-day-old wild-type flies (Figure S1A. a-a′′, Figure S1C). The number of mitotic ISCs with supernumerary centrosomes per gut was 0.8 in 45-day-old wild type flies and 5.75 in 10-day-old *Cat*^{n1} mutant flies, as compared to 0.05 in 10-day-old wild type flies (Figure S1D). These results indicate that with aging and subsequent to oxidative stress exposure, there is an increase in centrosome amplification in midgut ISCs. Interestingly, β-HB treatment reduces the age- and oxidative stress-related increase in the number of PH3-positive cells (Figure S1B). β-HB treatment reduces age- and oxidative stress-related increase of supernumerary centrosomes in 0.48% of mitotic ISCs in 45-day-old wild-type flies (Figure S1A d-d′′, Fig.S1C) and in 7.68% of mitotic ISCs in 10-day-old *Cat*^{n1} mutant flies (Figure S1A f-f′′, Figure S1C). The number of mitotic ISCs with supernumerary centrosomes per gut was reduced by 0.07 in 45-day-old wild type flies, and by 3.45 in 10-day-old *Cat*^{n1} mutant flies, whereas no change was observed in 10-day-old wild type flies (Figure S1D). To confirm whether β-HB inhibits oxidative stress-induced centrosome amplification in the midgut, we applied extrinsic oxidative stress. Briefly, three-day-old wild-type flies with or without 2 mM β-HB treatment for 6 days, were treated with 10 mM PQ for 18 h. Both mitotic ISCs (4.1 to 62.56, Figure S1A g-g′′, S1B,) and mitotic ISCs with supernumerary centrosomes (1.16% to 10.66%, Figure S1A g-g′′, S1C) were observed to increase in the PQ-treated wild type flies (Figure S1A g-g′′, S1B, S1C), as compared to control flies (Figure S1A a-a′′, S1B, S1C). Furthermore, there was an increase in the number of mitotic ISCs with supernumerary centrosomes per gut in the PQ-treated flies (0.05 to 6.28; Figure S1D). However, in β-HB pre-treated wild type flies, the number of PH3-positive cell (62.56 to 36.65), mitotic ISCs with supernumerary centrosomes (10.66% to 7.37%), and the number of mitotic ISCs with supernumerary centrosomes per gut (6.28% to 2.7%) were decreased after PQ treatment (Figure S1A h-h′′, S1B, S1C).

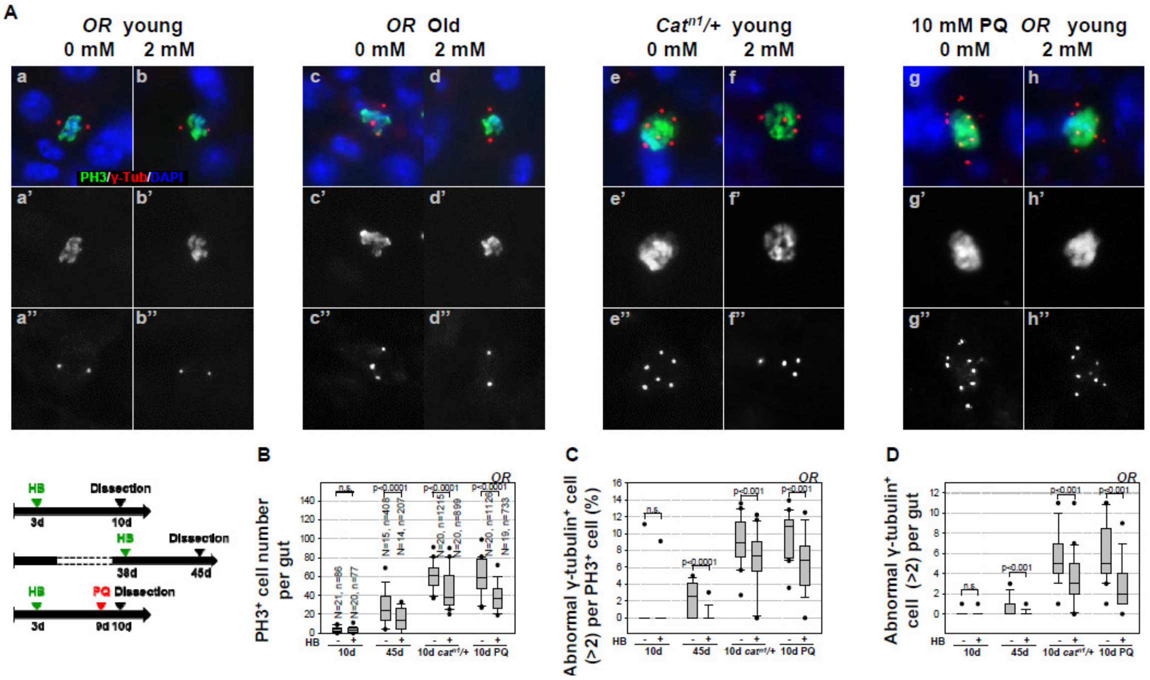

Figure S1. β-HB inhibits age- and oxidative stress-related centrosome amplification in midgut ISCs. (A) Guts from 10-day-old wild type flies (a–b″), 45-day-old wild type flies (c-d″), and 10-day-old Cat^{n1} mutant flies (e–f″), without (a–a″, c-c′′, e-e′′) or with (b-b′′, d-d′′, f-f′′) 2 mM β-HB feeding for seven days, were stained with anti-γ-tubulin (red), anti-PH3 (green), and DAPI (blue). Ten-day-old wild type flies, without (g–g″) or with (h–h″) 2 mM β-HB feeding for 6 days, were treated with 10 mM PQ in standard media for 20 h, after which their guts were stained with anti-γ-tubulin (red), anti-PH3 (green), and DAPI (blue). a′, b′, c′, d′, e′, f′, g′, and h′ indicate enlarged PH3 stained images. a″, b″, c″, d″, e″, f″, g″, and h″ indicate enlarged γ-tubulin stained images. Original magnification is 400×. (B) The number of PH3-positive cells was counted in whole guts from 10-day-old wild type, 45-day-old wild type, 10-day-old Cat^{n1} mutant, and 10-day-old PQ-treated wild type flies, with or without β-HB feeding for seven days. N represents the number of observed guts, and n is the number of observed PH3-positive cells. n.s. indicates not significant ($p>0.05$). (C) The frequency of supernumerary centrosomes (>2) per mitotic ISC in 10-day-old wild type, 45-day-old wild type, 10-day-old Cat^{n1} mutant, and 10-day-old PQ-treated wild type flies with or without β-HB feeding for seven days. The centrosome numbers were counted in mitotic ISCs (PH3-positive cells) in the midgut. n.s. indicates not significant ($p>0.05$). (D) The frequency of mitotic ISCs with supernumerary centrosomes per gut in 10-day-old wild type, 45-day-old wild type, 10-day-old Cat^{n1} mutant, and 10-day-old PQ-treated wild type flies with or without β-HB feeding for seven days. The error bar represents standard error. p-values were calculated using Student's *t*-test. n.s. indicates not significant ($p>0.05$).
